# Occupational medicine residency in Brazil: trends, positions, and regional distribution

**DOI:** 10.47626/1679-4435-2025-1398

**Published:** 2025-08-31

**Authors:** Claudio José dos Santos Júnior, Frida Marina Fischer

**Affiliations:** 1 School of Public Health, Universidade de São Paulo, São Paulo, SP, Brazil

**Keywords:** medicine, internship and residency, physicians’ distribution, occupational medicine, Brazil, medicina, internato e residência, distribuição de médicos, medicina do trabalho, Brasil

## Abstract

**Introduction:**

Resolution No. 1,634/2002 of the Brazilian Federal Board of Medicine (Conselho Federal de Medicina) and Resolution No. 5/2002 of the Brazilian National Medical Residency Commission (Comissão Nacional de Residência Médica) introduced the opportunity for occupational medicine training through medical residency programs. Nonetheless, recent studies analyzing the landscape of occupational medicine residency programs remain scarce, particularly with regard to their growth, geographical distribution, and occupancy rates.

**Objectives:**

To assess the increase in the number of graduates from occupational medicine residency programs, the regional distribution of these programs, the occupancy of available positions, and the proportion of specialists in relation to the workforce.

**Methods:**

This was a documentary study that used data from the Brazilian National Medical Residency Commission system and the Brazilian Federal Board of Medicine. Descriptive and temporal trend analyses were conducted for the 2006-2023 period.

**Results:**

There was a growing trend in the number of occupational medicine residency graduates in Brazil (annual percentage change = 3.32%; p = 0.012). Residency was offered in 8 out of the 27 Brazilian states; moreover, 60.7% of graduates completed their programs in the Southeast region, and 57% of the 94 positions available in 2023 were filled. The ratio of occupational physicians per 1,000 employed individuals was 0.178.

**Conclusions:**

An increase in occupational medicine residency graduates was identified, as well as unequal regional distribution and low occupancy of positions in Brazil.

## Abstract (pt)

**Introdução:**

A Resolução nº 1.634/2002 do Conselho Federal de Medicina e a Resolução nº 5/2002 da Comissão Nacional de Residência Médica introduziram a oportunidade de formação em medicina do trabalho por meio de programas de residência médica. No entanto, há escassez de estudos recentes que analisem o panorama dos programas de residência em medicina do trabalho, particularmente no que se refere ao seu crescimento, distribuição geográfica e taxas de ocupação.

**Objetivos:**

Avaliar o aumento do número de egressos de programas de residência em medicina do trabalho, a distribuição regional dos programas, a ocupação das vagas e a proporção de especialistas em relação à força de trabalho.

**Métodos:**

Trata-se de pesquisa documental que utilizou dados do Sistema da Comissão Nacional de Residência Médica e do Conselho Federal de Medicina. Foram realizadas análises descritivas e de tendência temporal para o período de 2006 a 2023.

**Resultados:**

Foi identificada tendência de crescimento no número de egressos da residência em medicina do trabalho no Brasil (variação percentual anual = 3,32%; p = 0,012). A residência era oferecida em 8 dos 27 estados do país; além disso, 60,7% dos egressos realizaram programas na região Sudeste, e 57% das 94 vagas disponíveis em 2023 foram ocupadas. A razão de médicos do trabalho por 1.000 pessoas ocupadas foi de 0,178.

**Conclusões:**

Identificou-se um aumento no número de egressos de residência em medicina do trabalho, distribuição regional desigual e baixa ocupação das vagas no Brasil.

## INTRODUCTION

Occupational medicine (OM) is a medical specialty dedicated to promoting workers’ health and safety. It encompasses the prevention, diagnosis, treatment, and rehabilitation of work-related illnesses, as well as the analysis and management of occupational risks.^1^ Occupational physicians, responsible for promoting the comprehensive health of workers in both the public and private sectors, play a central role in ensuring healthy, safe, and productive work environments, as well as in fostering well-being and quality of life in these settings.^2^

Resolution No. 1,634/2002 of the Brazilian Federal Board of Medicine (Conselho Federal de Medicina, CFM), which recognized 50 medical specialties, including OM, and Resolution No. 5/2002 of the Brazilian National Medical Residency Commission (Comissão Nacional de Residência Médica, CNRM) introduced the possibility of OM training through medical residency programs. Following this recognition, national educational and health care institutions structured OM residency programs in accordance with the legislation. As a result, in November 2003, six OM residency programs were authorized by the CNRM to begin activities in January 2004.^3^

The first cohort of occupational physicians trained through specific, direct-entry OM residency programs completed their specialization in 2006. Since then, several changes have occurred in the landscape of medical training in Brazil, including modifications to specialist training.^4^,^5^ Nonetheless, recent studies analyzing the landscape of OM residency programs remain scarce, particularly with regard to their expansion, geographical distribution, and occupancy rates.

Therefore, this study aimed to evaluate the increase in the number of graduates from OM residency programs, the regional distribution of these programs, the occupancy of available positions, and the proportion of specialists in relation to the employed Brazilian workforce.

## METHODS

This was a retrospective documentary study that analyzed the temporal trend, available positions, and the regional distribution of OM training in Brazil from 2006 to 2023.

Data were obtained from the CNRM System (SisCNRM). Additionally, data from the CFM regarding the number of physicians and occupational physicians with active registrations in 2023 were also examined. Estimates of the number of employed individuals aged ≥ 14 years (ie., the economically active population [EAP]) for the same period were also obtained from the Brazilian Institute of Geography and Statistics (Instituto Brasileiro de Geografia e Estatística).

Descriptive statistics were employed, including measures of absolute and relative frequency, along with temporal trend analysis using Prais-Winsten linear regression, as described by Antunes & Cardoso.^6^ For graphical visualization of the trend, a time series graph was prepared, employing a second-order moving average to smooth short-term data fluctuations.

Analyses were conducted using Stata 16 software, with a significance level of 5%.

Data were provided by the Ministry of Education through the CNRM and by the CFM’s Citizen Information Service via the Access to Information Law, with no need for review by a Research Ethics Committee.

## RESULTS

A total of 376 physicians completed OM residency programs over the 17-year period analyzed. Of these, 60.7% were from states in the Southeast region of Brazil. OM residency programs were offered in only 8 out of the 27 Brazilian states. The average number of graduates per year was 20.89 ± 5.17.

**Figure 1:**
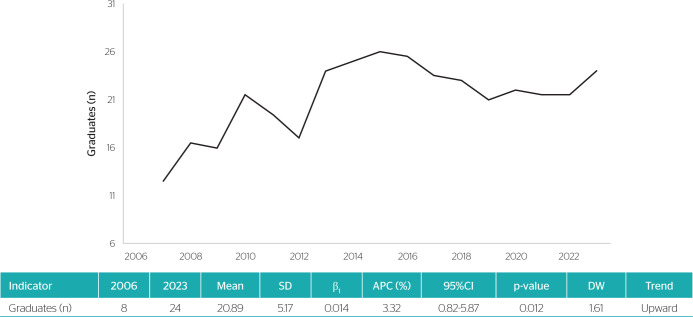
Temporal trend in the number of graduates from occupational medicine residency programs in Brazil, 2006-2023. APC = annual percentage change; DW = Durbin-Watson statistic; SD = standard deviation.

The temporal trend analysis revealed a growth pattern in the number of OM residency graduates in the Brazil between 2006 and 2023 (annual percentage change [APC] = 3.32%; 95%CI 0.82-5.87; p = 0.012) (Figure 1).

In 2023, 18 institutions offered OM residency programs, the majority of which (11) were located in the Southeast region. Of the 94 available positions that year, 54 (57%) were filled (Table 1). Also in 2023, there were 17,722 active occupational physicians in Brazil. The proportion of these professionals in relation to the total number of physicians was 4.33%. The ratio of OM specialists per 10,000 employed individuals was 1.78, compared to 4.10 per 1,000 employed individuals for physicians overall (Table 2).

**Table 1:** Institutions offering occupational medicine residency programs, and number of available and filled positions in Brazil, 2023

Municipality (state)	Institution	Positions				Occupancy (%)
		Available		Filled		
		R1	R2	R1	R2	
Maceió (AL)	Fundação Educacional Jayme de Altavila	2	2	2	2	100
Salvador (BA)	Hospital Professor Edgard Santos (Universidade Federal da Bahia)	7	7	3	4	50
Brasília (DF)	Federal District Health Department	2	2	2	3	125
Belo Horizonte (MG)	Hospital das Clínicas da Universidade Federal de Minas Gerais	4	4	4	3	88
Ipatinga (MG)	Hospital Marcio Cunha	2	2	0	2	50
Belo Horizonte (MG)	Hospital Municipal Odilon Behrens	1	1	0	0	0
Curitiba (PR)	Hospital do Trabalhador	2	2	0	2	50
Rio de Janeiro (RJ)	Hospital Universitário Pedro Ernesto University (Universidade do Estado do Rio de Janeiro)	2	2	2	0	50
Rio de Janeiro (RJ)	Instituto Nacional do Câncer	2	2	0	0	0
Porto Velho (RO)	Centro de Ensino São Lucas Ltda.	2	2	0	0	0
Porto Alegre (RS)	Hospital de Clínicas de Porto Alegre	4	4	4	1	63
Campinas (SP)	School of Medical Sciences (Universidade de Campinas)	4	4	4	3	88
São Paulo (SP)	School of Medicine (Universidade de São Paulo)	2	2	2	2	100
Santo André (SP)	ABC School of Medicine	2	2	2	2	100
São Paulo (SP)	Hospital Alemão Oswaldo Cruz	2	2	2	0	50
São Paulo (SP)	Hospital do Servidor Público Estadual Francisco Morato Oliveira	2	2	0	0	0
São Paulo (SP)	Irmandade da Santa Casa de Misericórdia de São Paulo	3	3	1	2	50
Piracicaba (SP)	Município de Piracicaba	2	2	0	0	0

AL = Alagoas; BA = Bahia; DF = Distrito Federal; MG = Minas Gerais; PR = Paraná; R1 = first year of residency; R2 = second year of residency; RJ = Rio de Janeiro; RO = Rondônia; RS = Rio Grande do Sul; SP = São Paulo.

**Table 2:** Occupational physicians and physicians with active registration, by state, and their ratios in relation to the economically active population in Brazil, 2023

State	OP	MD	EAP*	OP/MD (%)	OP/10,000 EAP ratio^†^	MD/1,000 EAP ratio^‡^
Acre	30	790	319	3.80	0.94	2.48
Alagoas	200	3,986	1,236	5.02	1.62	3.22
Amazonas	206	3,640	1,770	5.66	1.16	2.06
Amapá	16	749	385	2.14	0.42	1.95
Bahia	796	18,153	6,138	4.38	1.30	2.96
Ceará	298	12,408	3,661	2.40	0.81	3.39
Distrito Federal	542	16,257	1,605	3.33	3.38	10.13
Espírito Santo	686	10,621	2,038	6.46	3.37	5.21
Goiás	531	14,174	3,789	3.75	1.40	3.74
Maranhão	134	4,686	2,674	2.86	0.50	1.75
Minas Gerais	2,688	4,9312	10,620	5.45	2.53	4.64
Mato Grosso do Sul	186	6,144	1,437	3.03	1.29	4.28
Mato Grosso	270	5,832	1,812	4.63	1.49	3.22
Pará	300	6,066	3,797	4.95	0.79	1.60
Paraíba	201	6,362	1,536	3.16	1.31	4.14
Pernambuco	420	12,437	3,647	3.38	1.15	3.41
Piauí	93	4,126	1,299	2.25	0.72	3.18
Paraná	1,004	27,532	5,902	3.65	1.70	4.66
Rio de Janeiro	1,352	38,316	7,996	3.53	1.69	4.79
Rio Grande do Norte	185	4,539	1,346	4.08	1.37	3.37
Rondônia	71	2,284	833	3.11	0.85	2.74
Roraima	19	811	251	2.34	0.76	3.23
Rio Grande do Sul	1,734	24,227	5,843	7.16	2.97	4.15
Santa Catarina	730	18,913	3,984	3.86	1.83	4.75
Sergipe	177	3,671	976	4.82	1.81	3.76
São Paulo	4,787	111,098	24,199	4.31	1.98	4.59
Tocantins	66	2,217	747	2.98	0.88	2.97
Brazil	17,722	409,351	99,840	4.33	1.78	4.10

EAP = economically active population (individuals ≥ 14 years old who were employed) in thousands of inhabitants, 3rd quarter of 2023; MD = physicians; OP = occupational physicians.

† Employed occupational physicians per 10,000 employed physicians.

‡ Employed physicians per 1,000 overall physicians.

## DISCUSSION

Between 2006 and 2023, there was an upward trend in the number of graduates from OM residency programs in Brazil. Approximately 21 residents graduated annually over the 17 years analyzed. This trend aligns with the overall growth in the number of medical specialists and residency positions in Brazil, estimated at 84% over the last decade.^5^,^7^

OM residency programs were offered in only 29.6% of the 27 Brazilian states, with most active programs and residency positions concentrated in the Southeast region. This geographic concentration highlights disparities in the availability of specialized training in OM. This phenomenon reflects broader spatial inequalities in the distribution of medical school seats, although it is not limited to generalist medical education.^8^,^9^

One factor contributing to this scenario is the requirement for specialized personnel and well-equipped facilities to establish a residency program, which is often unfeasible in remote cities or areas lacking the necessary infrastructure for specialist training. Article 10 of CNRM Resolution No. 5/2002 specifies that institutions offering OM residency programs must meet stringent criteria regarding facilities, equipment, and organizational capacity to ensure the proper development of specialist training programs.^10^

Chaves et al.^11^ observed that the Southeast states, due to their large populations, account for more than half of all active specialists in the country. In OM, this concentration is further influenced by the Southeast’s industrial and economic prominence.^12^,^13^ The region’s intense industrial and commercial activity demands a significant number of occupational physicians to coordinate occupational health medical programs, integrate specialized safety and OM services, and conduct worker health interventions. The Southeast also accounts for the highest absolute number of work-related accidents and illnesses in Brazil.^14^

Only 57% of OM residency positions were filled in 2023, suggesting low demand for these programs and possibly indicating limited interest in residency-level OM training. Other barriers include low stipend amounts and the limited emphasis on OM in medical school curricula.^15^-^20^ A recent study showed that OM had the lowest average consultation fees among all medical specialties analyzed, placing OM in an unfavorable position compared to 29 other specialties.^21^

A national study on inequality in the distribution of the medical workforce identified key challenges to hiring specialists, including low remuneration, inadequate technical resources, excessive workload, and a shortage of certified or experienced professionals.^22^ These challenges reflect broader difficulties faced by occupational physicians in Brazil.

The ratio of occupational physicians per 1,000 employed individuals was 0.178, compared to 4.10 for overall physicians. Despite this disparity, OM remains the 7th largest medical specialty in Brazil, following internal medicine, pediatrics, general surgery, obstetrics and gynecology, anesthesiology, and orthopedics and traumatology.^7^

Silveira & Dias noted that revisions to Regulatory Standard No. 7 in the 1990s expanded the scope of OM and let to the proliferation of OM specialization courses, which were predominantly theoretical.^3^ However, according to CFM Resolution No. 2,148/2016, these courses no longer grant specialist titles nor qualify physicians for the specialist certification exam.^23^

This resolution allows physicians without residency training to take the certification exam if they demonstrate professional experience equivalent to twice the duration of the residency program.^23^ This policy broadens the pathway to specialist certification, especially in regions without residency programs, and contributes to the diversity and qualification of Brazil’s OM workforce.

Nonetheless, the current annual output of approximately 21 OM residency graduates is insufficient to meet the growing demand for specialists in a country as large as Brazil. Recent advancements, such as CNRM Resolution No. 13/2019, established a competency-based framework for OM residency programs.^24^ While this framework ensures quality standards, significant challenges persist, including regional disparities, insufficient infrastructure, low program occupancy rates, discouraging remuneration, and a lack of recognition within the health care system.

Addressing these issues requires coordinated efforts among health care institutions, regulatory bodies, and professional organizations to improve working conditions for occupational physicians, elevate the value of the specialty, and expand equitable access to high-quality training.

## CONCLUSIONS

The findings of this study reveal an upward trend in the number of OM residency graduates, as well as significant regional disparities (with most programs concentrated in Southeastern Brazil), low occupancy of available positions, and a pressing need to strengthen the specialty to meet the demands of Brazil’s workforce.
